# Azole Antifungal Resistance in *Candida albicans* and Emerging Non-*albicans Candida* Species

**DOI:** 10.3389/fmicb.2016.02173

**Published:** 2017-01-12

**Authors:** Sarah G. Whaley, Elizabeth L. Berkow, Jeffrey M. Rybak, Andrew T. Nishimoto, Katherine S. Barker, P. David Rogers

**Affiliations:** ^1^Department of Clinical Pharmacy, College of Pharmacy, University of Tennessee Health Science CenterMemphis, TN, USA; ^2^Center for Pediatric Pharmacokinetics and Therapeutics, University of Tennessee Health Science CenterMemphis, TN, USA

**Keywords:** antifungal, azole, resistance, *Candida albicans*, *Candida parapsilosis*, *Candida glabrata*, *Candida tropicalis*, *Candida krusei*

## Abstract

Within the limited antifungal armamentarium, the azole antifungals are the most frequent class used to treat *Candida* infections. Azole antifungals such as fluconazole are often preferred treatment for many *Candida* infections as they are inexpensive, exhibit limited toxicity, and are available for oral administration. There is, however, extensive documentation of intrinsic and developed resistance to azole antifungals among several *Candida* species. As the frequency of azole resistant *Candida* isolates in the clinical setting increases, it is essential to elucidate the mechanisms of such resistance in order to both preserve and improve upon the azole class of antifungals for the treatment of *Candida* infections. This review examines azole resistance in infections caused by *C. albicans* as well as the emerging non-*albicans Candida* species *C. parapsilosis, C. tropicalis, C. krusei*, and *C. glabrata* and in particular, describes the current understanding of molecular basis of azole resistance in these fungal species.

## Introduction

*Candida albicans* and emerging non-*albicans Candida* (NAC) species such as *C. glabrata, C. parapsilosis, C. tropicalis*, and *C. krusei* can cause superficial infections of the oral and vaginal mucosa as well as disseminated bloodstream and deep-tissue infections. Species involvement varies by infection site and by geography. *Candida* infections are most often caused by *C. albicans* as evidenced by epidemiological studies in the United States (Cleveland et al., 2015), Europe (Klingspor et al., 2015), and the Middle East (Sharifzadeh et al., 2013). Of all the NAC species, *C. glabrata* is the most commonly isolated from patients with candidemia in North America (Sobel, 2010; Azie et al., 2012; Pfaller et al., 2014b), and Northern Europe (Lortholary et al., 2014; Milazzo et al., 2014), as well as other geographic areas studied with the exception of Latin America (Pfaller et al., 2010). *C. glabrata* is also the most common NAC species found to be the causative agent in vulvovaginal candidiasis (VVC) (Corsello et al., 2003; Holland et al., 2003; Richter et al., 2005; Vermitsky et al., 2008; Mahmoudi Rad et al., 2012) and candiduria (Sobel et al., 2000; Kauffman, 2005). In some patient populations, for example, candidemia in patients with hematologic malignancy and VVC in diabetic patients, *C. glabrata* is even more common than *C. albicans* (Goswami et al., 2006; Ray et al., 2007; Hachem et al., 2008). *C. parapsilosis* is well known for its threat to the pediatric population, as it is responsible for 17–50% of all fungemia in infants and neonates (Abi-Said et al., 1997; Krcmery et al., 1999). *C. parapsilosis* is also second only to *C. albicans* in incidence as a cause of *Candida* endocarditis with mortality rates between 42 and 65% (Weems, 1992; Garzoni et al., 2007). In the Asia-Pacific region, *C. tropicalis* has been reported to constitute 20–45% of *Candida* isolates (Kothari and Sagar, 2009; Pfaller et al., 2010). *C. tropicalis* infections are commonly associated with malignancy, with some studies reporting higher prevalence among patients with hematologic diseases such as acute myeloid leukemia (Weinberger et al., 2005; Nucci and Colombo, 2007; Tang et al., 2014, 2015; Cornely et al., 2015). Mortality associated with *C. tropicalis* candidemia in these populations unfortunately remains high, ranging from 30 to 70%, with the highest rates most commonly observed among the elderly (Weinberger et al., 2005; Nucci and Colombo, 2007; Morii et al., 2014; Cornely et al., 2015; Wang et al., 2015). *C. krusei* is the fourth most common NAC species associated with invasive candidiasis and candidemia, accounting for approximately 2.7% of NAC species isolated across the United States (Pfaller et al., 2014b). Moreover, the number of *C. krusei* isolates implicated in these types of infections has increased over time (Pfaller et al., 2014a,b). In particular, patients with hematologic malignancies and bone marrow transplants have been shown to be at increased risk of *C. krusei* infection (Merz et al., 1986; Wingard et al., 1991; Pfaller et al., 2008).

## Azole resistance in *Candida* infections

There are several classes of compounds that comprise the arsenal used to treat *Candida* infections. The polyenes, azoles, echinocandins, nucleoside analogs, and allylamines are used with varying efficacy depending on the type and site of infection and the sensitivity of the *Candida* species (Pfaller et al., 2010; Pfaller and Diekema, 2012b; Pfaller et al., 2013; Pappas et al., 2016). The most commonly prescribed antifungal used for most *C. albicans* infections is fluconazole, a member of the azole class of antifungals (Pfaller et al., 2010). Azoles inhibit 14-α-sterol demethylase, encoded by the *ERG11* gene, which is an enzyme involved in the biosynthesis of the fungal-specific membrane sterol ergosterol. As some NAC species exhibit intrinsic resistance to azoles, their use is likely a contributing factor to the more frequent incidence of infections caused by these NAC species (Oxman et al., 2010; Lortholary et al., 2011; Fothergill et al., 2014). Moreover, many studies have documented the ability of *Candida* to develop high-level resistance to azole antifungals (Oxman et al., 2010; Lortholary et al., 2011). A compilation of fluconazole MIC ranges and epidemiological cutoff values for *Candida* species is presented in Table 1.

**Table 1:** **Fluconazole MIC ranges and epidemiological cutoff values for ***Candida*** species**.

***Candida*** **species (# of isolates tested)**	**MIC range^1^ (mode)**	**Percent of resistant isolates**
*C. albicans* (5265)	0.06 – ≥128 (0.12)	3.5
*C. glabrata* (7538)	0.12 – ≥128 (4)	7.8
*C. krusei* (1075)	0.25 – ≥128 (16)	96.6
*C. parapsilosis* (6023)	0.06 – ≥128 (0.5)	3.4
*C. tropicalis* (3748)	0.06 – ≥128 (0.25)	2.3

*All values are in mg/L*.

*(Clinical and Laboratory Standards Institute, 2012; Espinel-Ingroff et al., 2014)*.

Infections caused by *C. albicans* are associated with varying levels of fluconazole resistance depending on the type of infection. *C. albicans* isolates from candidemic patients have the lowest incidence of azole resistance (0–5%) (Diekema et al., 2012; Pfaller et al., 2015; Ying et al., 2015). The incidence of fluconazole resistance in *C. albicans* isolates from oropharyngeal candidiasis (OPC) is higher and depends upon previous fluconazole treatment and prior OPC infections (Enwuru et al., 2008; Berberi et al., 2015). *C. glabrata* has the highest incidence of azole resistance among *Candida* clinical isolates and exhibits intrinsic decreased susceptibility to the azole class of antifungals (Oxman et al., 2010; Pfaller et al., 2014b), including the newest addition to the class, isavuconazole (Castanheira et al., 2014). *C. glabrata* is also able to develop high-level resistance after exposure to azole antifungals (Fidel et al., 1999; Lee et al., 2009) and is one of the most frequent species isolated in breakthrough infections from patients receiving azole prophylaxis (Bennett et al., 2004; Imhof et al., 2004; Hachem et al., 2008). Of increasing concern are the number of multidrug resistant isolates of *C. glabrata* that are being recovered clinically (Manzano-Gayosso et al., 2003; Chapeland-Leclerc et al., 2010; Hull et al., 2012; Pfaller et al., 2012a; Cho et al., 2015). In the Asia-Pacific region, fluconazole resistance in *C. tropicalis* ranges from 0 to as high as 83% (Yang et al., 2004, 2008; Yoo et al., 2009). The worldwide incidence of fluconazole resistance in *C. parapsilosis* disseminated infections ranges between 2 and 5% (Chen et al., 2006; Martí-Carrizosa et al., 2014; Pfaller et al., 2015). As *C. krusei* exhibits intrinsic resistance to fluconazole, there is some controversy whether its increased infection rate is related to fluconazole prophylaxis or previous treatment (Hope et al., 2002; Lin et al., 2005; Blot et al., 2006; Gong et al., 2016). Clearly, an understanding of molecular mechanisms driving intrinsic and development of high-level azole resistance is warranted.

## Azole antifungal resistance mechanisms

### Candida albicans

**Figure 1:**
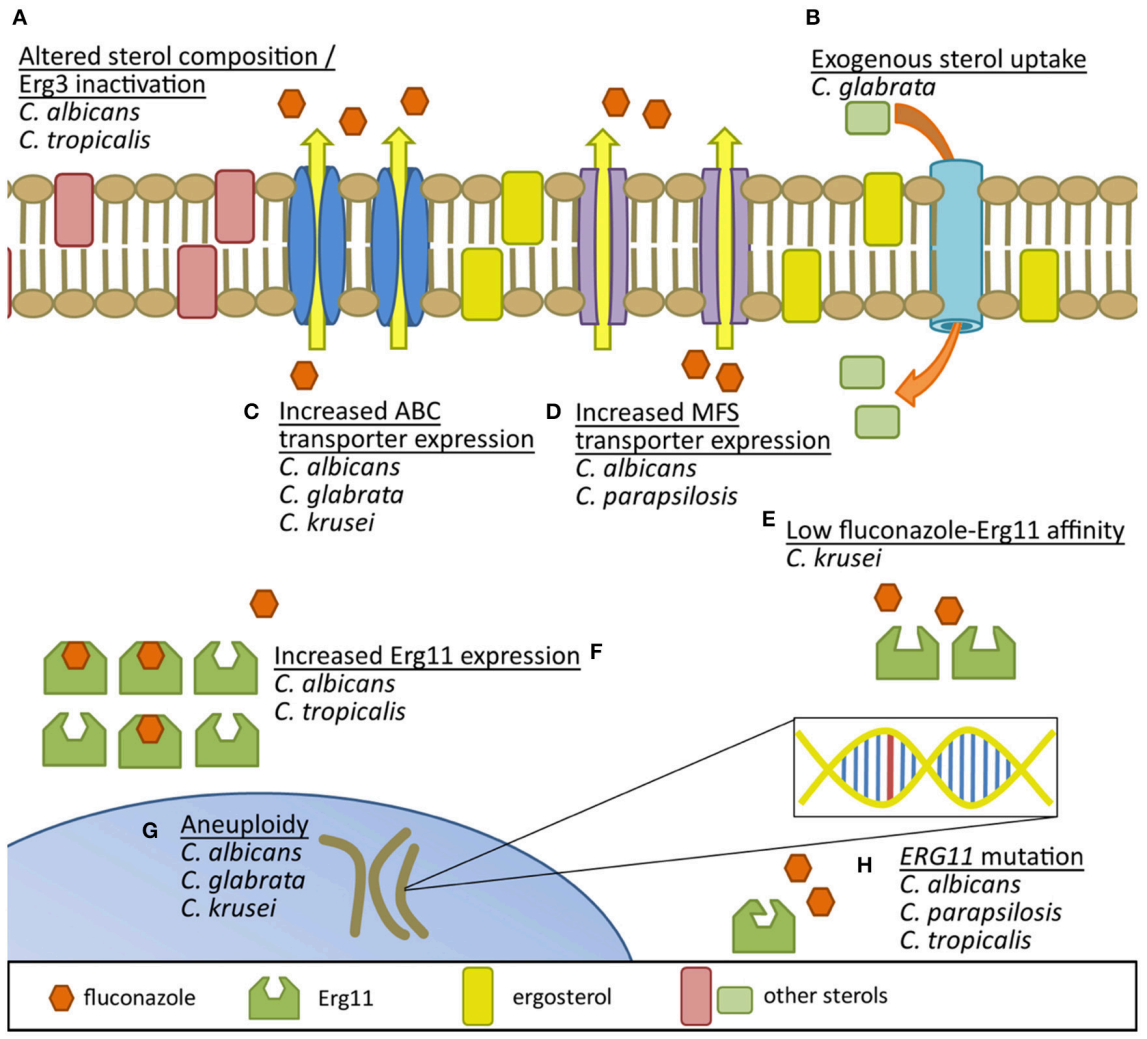
**Comparison of documented fluconazole resistance mechanisms in ***Candida*** species. (A)** Erg3 inactivation results in utilization of alternative sterols in the yeast membrane. **(B)** Uptake of exogenous sterols helps circumvent endogenous sterol production inhibition by fluconazole. Increased production of both **(C)** ATP-binding cassette efflux pumps and **(D)** major facilitator superfamily transporters reduces intracellular accumulation of azoles. **(E)** Inherently low affinity of fluconazole binding to species-specific Erg11 may decrease fluconazole's potential to inhibit the protein. **(F)** Increased expression of Erg11 protein can help overcome azole activity and **(G)** aneuploidy may promote genetic adaptation to azole exposure. **(H)** Mutations in *ERG11* can also result in proteins with reduced affinity for fluconazole binding.

Resistance to azole antifungals in *Candida* (summarized in Figure 1) has been most extensively studied in *C. albicans*. One mechanism of resistance identified in this species is the presence of point mutations in *ERG11*. Previous studies have identified amino acid substitutions that result in decreased fluconazole susceptibility and have noted that several of these critical allelic variations cluster in three “hot spot” regions within Erg11p (Marichal et al., 1999). Recently, 63 fluconazole-resistant *C. albicans* clinical isolates were examined for mutations within their *ERG11* alleles, and 55 were found to carry at least one mutation that resulted in amino acid substitutions, with nine such predicted amino acid substitutions being novel (Flowers et al., 2015). Molecular modeling of the substitutions that resulted in decreased fluconazole susceptibility when expressed in a susceptible background revealed that the mutations clustered in either the predicted catalytic site, the fungus-specific external loop, or on the proximal surface potentially interacting with the loop or near the heme. Additionally, a study involving site-directed mutagenesis of wild-type *ERG11* to introduce mutations identified in 23 *C. albicans* clinical isolates demonstrated nine of these mutations result in increased fluconazole resistance (Xiang et al., 2013). Five of the amino acid substitutions were predicted to be at or near the active site of Erg11p.

Another mechanism of fluconazole resistance in *C. albicans* is the increased expression of *ERG11* due to activating mutations in the gene encoding the zinc-cluster transcriptional regulator Upc2p. *C. albicans* Upc2 is a homolog of the *Saccharomyces cerevisiae ERG* gene regulator pair Upc2/Ecm22. Initially, Upc2 involvement in fluconazole resistance in *C. albicans* was demonstrated when Δ*upc2 C. albicans* strains were shown to be highly susceptible to azoles while those over-expressing Upc2 had increased fluconazole resistance (MacPherson et al., 2005). Further studies examining a matched set of fluconazole-susceptible and—resistant *C. albicans* clinical isolates in which fluconazole resistance was not associated with overexpression of drug efflux pumps revealed SNPs in one *UPC2* allele and overexpression of several *ERG* genes and *UPC2* in the resistant isolate (Dunkel et al., 2008a). Expression of *UPC2* alleles in fluconazole-susceptible strains resulted in increased fluconazole resistance (Dunkel et al., 2008a; Heilmann et al., 2010; Hoot et al., 2011). Interestingly, three additional matched sets of *ERG11*-overexpressing clinical *C. albicans* isolates have been described which have no sequence differences in *UPC2* between the susceptible and resistant isolates in each pair (Heilmann et al., 2010), indicating that other mechanisms of *ERG11* upregulation exist. While these studies were important in establishing Upc2p as a regulator of *ERG11* expression in the context of fluconazole resistance, it was assumed that *UPC2*-mediated fluconazole resistance is a rare occurrence. However, a large study involving 63 fluconazole-resistant *C. albicans* clinical isolates demonstrated 47 of these isolates overexpressed *ERG11* by at least 2-fold (Flowers et al., 2012). Twenty-nine of these *ERG11*-overexpressing isolates contained a missense mutation in *UPC2*, and eight single amino acid substitutions were elucidated from their *UPC2* alleles. Seven of these alleles were found to be associated with increased *ERG11* expression, increased ergosterol production, and decreased fluconazole susceptibility.

Two other mechanisms of fluconazole resistance in *C. albicans* involve the overexpression of drug efflux pumps Mdr1p and Cdr1p/Cdr2p. *TAC1* (transcriptional activator of C*DR* genes) is a zinc-cluster transcription factor whose regulon is hallmarked by the ATP-binding cassette (ABC) transporter-encoding genes *CDR1* and *CDR2* (Coste et al., 2004). Activation of expression of the *TAC1* regulon is through binding of *TAC1* to the DRE (drug response element) present in the promoters of *TAC1*-regulated genes (Coste et al., 2004; Liu et al., 2007). At least nine hyperactive *TAC1* alleles have been identified (Coste et al., 2007), and fluconazole minimum inhibitory concentrations (MIC) associated with the isolates from which these alleles have been discovered have revealed that *TAC1* demonstrates codominance resulting in intermediate fluconazole MIC in *TAC1*-heterologous strains and high fluconazole MIC upon loss of heterozygosity (Coste et al., 2006, 2007). Because *TAC1* resides on the left arm of Chr5 with *ERG11*, such loss of heterozygosity in the presence of hyperactive *TAC1* and mutated *ERG11* results in high-level azole resistance (Coste et al., 2007; Selmecki et al., 2008).

Mdr1p is a major facilitator superfamily (MFS) efflux pump usually expressed at non-detectable levels in wildtype *C. albicans* strains, induced in the presence of benomyl, diamide, and hydrogen peroxide, and constitutively overexpressed in some fluconazole-resistant *C. albicans* isolates (Alarco and Raymond, 1999). *MRR1*, multidrug resistance regulator 1, was identified by comparing the transcriptomes of sets of matched isolates in which the fluconazole-resistant isolates overexpressed *MDR1* (Morschhäuser et al., 2007). Disruption of *MRR1* in these resistant isolates led to a decrease in fluconazole MIC, while introduction of each of the mutant alleles individually into a wildtype fluconazole-susceptible background in the native *MRR1* locus conferred fluconazole resistance to the constructed strain. Another study examined additional *MRR1* allelic variations in *MDR1*-mediated fluconazole resistance (Dunkel et al., 2008b). In most cases the resistant isolates/strains were homozygous for the *MRR1* allele containing the gain-of-function mutations due to mitotic recombination and chromosome loss.

A less common mechanism of azole resistance in *C. albicans* is inactivation of the *ERG3* gene, which encodes the ergosterol biosynthesis enzyme sterol Δ^5,6^ desaturase. Erg3p catalyzes one of the final steps in the pathway and also converts nontoxic 14α-methylated sterol intermediates, that accumulate during azole treatment, into the toxic sterol 14α-methylergosta-8,24(28)-dien-3β,6α-diol. Inactivation or deletion of the *ERG3* gene, therefore, prevents such toxic sterols from being synthesized. Only a handful of clinical *C. albicans* isolates have documented azole resistance due to *ERG3* inactivation (Kelly et al., 1997; Nolte et al., 1997; Miyazaki et al., 1999; Chau et al., 2005; Martel et al., 2010; Morio et al., 2012).

Aneuploidy plays a role in azole resistance in *C. albicans* as demonstrated by comparative genome hybridization (Selmecki et al., 2006). As alluded to earlier, a common aneuploidy found in azole-resistant strains involves Chr5. Similarly, loss of heterozygosity (LOH) has been shown to occur in azole-resistant *C. albicans* (Coste et al., 2006). Examination of *TAC1* in a matched set of azole-susceptible and—resistant *C. albicans* isolates revealed that the susceptible isolate harbored two wildtype alleles of *TAC1*, while the resistant isolate contained only one of those alleles in which a single nucleotide polymorphism (SNP) translated into an activating amino acid substitution (N977D).

### Candida parapsilosis

Because azole resistance has been extensively studied in *C. albicans*, attempts to elucidate mechanisms of azole resistance in *C. parapsilosis* have involved examining orthologous genes and yielded mixed results. A study of a series of six isolates from a single patient found a single SNP in *MRR1* present in the two fluconazole-resistant isolates (Zhang et al., 2015). Nine fluconazole-resistant isolates were obtained from candidemia patients in a Brazilian hospital and examined for *CDR1, MDR1*, and *ERG11* overexpression as well as the presence of SNPs in the *ERG11* gene (Souza et al., 2015). Each of the resistant isolates possessed a single homozygous SNP (A395T) which corresponds to a Y132F amino acid substitution. In addition, while none of the isolates overexpressed *MDR1* as compared to the *C. parapsilosis* reference strain ATCC22019, *CDR1* expression was between 3.3- and 9.2-fold higher in these isolates as compared to the reference strain, and *ERG11* was overexpressed between 1.5- and 7.4-fold. While this study indicated an association between *CDR1* and *ERG11* and fluconazole resistance in *C. parapsilosis*, a causal link was not definitively proven.

In a larger-scale study, 30 resistant isolates, 37 susceptible-dose-dependent isolates, and 55 susceptible isolates were collected from hospitals in four U.S. cities, and their *ERG11* genes were sequenced (Grossman et al., 2015). Five SNPs were identified in 54 of the isolates; amino acid substitution Y132F, found in 17 resistant isolates, was the only one found exclusively in resistant isolates. Twenty-three isolates harbored SNPs in *MRR1*. Of the nine SNPs identified, only three were found exclusively in resistant isolates. Quantitative PCR measuring relative *MDR1* expression revealed nine isolates (six with a SNP in *MRR1*, three without) with at least 5-fold increase in *MDR1* expression compared to a composite expression level from a subset of susceptible isolates. However, the expression levels were a fraction of the levels achieved by *MDR1*-mediated azole resistance in *C. albicans*. Without definitive experiments in which introduction of a mutated *ERG11* allele confers azole resistance in a susceptible isolate, these results remain suggestive.

In an effort to identify potential mechanisms of azole resistance on a genome-wide scale in *C. parapsilosis*, fluconazole-, voriconazole-, and posaconazole-resistant strains were developed experimentally by serial passage in liquid culture containing either fluconazole, voriconazole, or posaconazole (Silva et al., 2011). The fluconazole- and voriconazole-resistant strains were cross-resistant to both fluconazole and voriconazole and possessed similar transcriptional profiles as assessed by microarray analysis; however, the posaconazole-resistant strain was not cross-resistant to the other azoles and had a distinct transcriptional profile. Among the genes differentially expressed in fluconazole- and voriconazole-resistant strains were the stress response gene *GRP2*, as well as *MDR1* and *MRR1*. *ERG11* was not differentially expressed in these strains. However, in the posaconazole-resistant strain, the ergosterol biosynthesis genes *ERG11* and *ERG6*, as well as *ERG* gene regulator *UPC2* were among the genes differentially expressed.

In a study using laboratory strains of *C. parapsilosis* in which previously-determined gain-of-function alleles of *CpMRR1* were introduced into the native locus, strains containing Mrr1p with a G583R amino acid substitution from a fluconazole-resistant *C. parapsilosis* isolate led to resistant fluconazole and voriconazole MIC compared to strains harboring the wildtype allele (Branco et al., 2015). Similarly, strains with single SNP-containing *MRR1* alleles had a ~5-fold increase in *MRR1* gene expression and ~70-fold increase in *MDR1* gene expression.

In another study, 35 unrelated fluconazole-resistant and four unrelated susceptible isolates of *C. parapsilosis* were examined to elucidate mechanisms of fluconazole resistance in *C. parapsilosis* (Berkow et al., 2015). Sixteen resistant isolates overexpressed *CDR1*, three other resistant isolates exhibited *MDR1* overexpression, and eight resistant isolates demonstrated overexpression of *ERG11* as compared to the susceptible isolates. When sequencing orthologues of *UPC2, MRR1*, and *TAC1* in order to identify putative gain-of-function mutations that would lead to overexpression of *ERG11, MDR1*, and *CDR1*, only one heterozygous mutation in *UPC2* was recovered from one isolate, suggesting that *ERG11* overexpression in fluconazole-resistant *C. parapsilosis* is not mediated by *UPC2*. *TAC1* mutations that were recovered did not fully correspond with *CDR1* overexpression and those recovered were not analogous to those found in gain-of-function *CaTAC1* alleles. Similarly, *MRR1* mutations recovered did not correspond to any mutations found in gain-of-function alleles of *CaMRR1*. Subsequently, *CDR1* was deleted from three of the *CDR1*-overexpressing isolates which only resulted in a one-dilution decrease in fluconazole MIC. *MDR1* deletion in three *MDR1*-overexpressing isolates revealed a one-dilution decrease in fluconazole MIC in two isolates and no change in fluconazole MIC in the third. To address the role of alterations in the ergosterol biosynthesis pathway in azole resistance in *C. parapsilosis, ERG11*, and *ERG3* were sequenced. No *ERG3* mutations were recovered, which was supported by the sterol profiles of the isolates. A single *ERG11* mutation (Y132F) was recovered in one resistant isolate and a combination of Y132F and R398I mutations was found in an additional ten isolates. In nine of these eleven isolates there was a change in the sterol profile indicative of a change in Erg11 functionality. This study indicates that while differential expression of efflux pumps is commonly found in azole-resistant *C. parapsilosis* isolates, the resistant phenotype is not solely due to their overexpression but instead is multifactorial and involves *ERG11* mutation and/or overexpression.

### Candida tropicalis

As compared with other species of *Candida*, relatively little is known about the mechanisms of azole resistance in *C. tropicalis*. An analysis of 52 clinical *C. tropicalis* isolates from China found the average *ERG11* expression level more than 4-fold higher among fluconazole-resistant isolates than -susceptible isolates (Jiang et al., 2013). Moreover, *ERG11* expression was even higher among a subset of fluconazole-resistant isolates also resistant to itraconazole and voriconazole. These results were recently echoed by a similar study characterizing 35 *C. tropicalis* isolates from Korean university hospitals, nine of which were fluconazole-non-susceptible (Choi et al., 2016). While considerable variability in *ERG11* expression (~150-fold) was observed in the highly fluconazole-susceptible group, *ERG11* expression was significantly higher among both less fluconazole-susceptible (MIC 1–2 μg/ml) and fluconazole-non-susceptible (MIC ≥ 4 μg/ml) isolates. This study also sequenced the *C. tropicalis UPC2* gene and found several heterozygous and homozygous mutations. However, many of these mutations have been observed in fluconazole-susceptible isolates not found to overexpress *ERG11*, and further characterization of their impact on the regulatory function of *UPC2* is needed.

Molecular characterization of azole-resistant clinical *C. tropicalis* isolates has also revealed alterations in the ergosterol biosynthetic pathway (Vandeputte et al., 2005; Eddouzi et al., 2013; Jiang et al., 2013; Choi et al., 2016). A fluconazole-resistant *C. tropicalis* isolate recovered from a clinical blood specimen from Tunisia was found to have mutations in both *ERG3* and *ERG11* which were individually observed to be detrimental to ergosterol biosynthesis when heterologously expressed in *S. cerevisiae* (Eddouzi et al., 2013). Notably, the *ERG11* mutation in this isolate consisted of a deletion of 132 nucleotides resulting in a D275V amino acid substitution and the loss of 44 amino acids near the N-terminus of Erg11p. Homozygous replacement of the wild-type *C. tropicalis ERG11* with the truncated clinical variant, with or without the associated clinical *ERG3* mutation, resulted in high-level fluconazole resistance in a fluconazole-susceptible reference strain of *C. tropicalis*. Additionally, an *ERG11* mutation resulting in decreased fluconazole susceptibility due to the amino acid substitution Y132F, has been well characterized in *C. albicans* and was recently observed in a fluconazole-resistant *C. tropicalis* isolate from a patient with candidemia (Tan et al., 2015).

One of the first studies to associate the overexpression of efflux pumps with azole resistance in *C. tropicalis* utilized serial passaging of a reference *C. tropicalis* isolate on media containing various concentrations of fluconazole to produce genetically-related isolates with reduced fluconazole susceptibility (Barchiesi et al., 2000). After passaging, all isolates with reduced susceptibility to fluconazole demonstrated increased expression of both *C. tropicalis MDR1* and a gene with high homology to *C. albicans CDR1*. In both cases, the increased expression was found to then be diminished in fluconazole-susceptible revertants obtained from further passaging on fluconazole-free media. The role of efflux pump overexpression in azole resistance among clinical *C. tropicalis* isolates has been less clearly defined. When the expression of *MDR1* and *CDR1* was examined in the aforementioned 52 clinical *C. tropicalis* isolates from China, no significant difference was observed between fluconazole-susceptible and -resistant isolates (Jiang et al., 2013). In contrast, among the 35 clinical isolates from Korean university hospitals, expression of both *MDR1* and *CDR1* was observed to be significantly higher among both less-fluconazole-susceptible and fluconazole-non-susceptible isolates. However, it is important to note the large degree of variability in the expression of *MDR1* and *CDR1* observed in the highly fluconazole-susceptible control group, ~50-fold and ~30-fold respectively (Wang et al., 2015). To date, experiments to directly delineate the potential role of these efflux pumps has yet to be performed in *C. tropicalis*, and the homologs of *C. albicans MRR1* and *TAC1* have not been examined.

### Candida krusei

*C. krusei* is intrinsically resistant to fluconazole, though the precise mechanism is not completely understood. Several studies have attributed *C. krusei*'s innate azole resistance to efflux pump activity, namely through the ATP-binding cassette transporter Abc1p, and reduced drug accumulation (Marichal et al., 1995; Katiyar and Edlind, 2001; Lamping et al., 2009) in combination with reduced azole affinity for Erg11p (Marichal et al., 1995; Venkateswarlu et al., 1997; Orozco et al., 1998; Guinea et al., 2006; Lamping et al., 2009). Changes in the cell membrane affecting membrane fluidity may be implicated in azole resistance as well since there is evidence to suggest that intracellular azole accumulation occurs through one or possibly both mechanisms of passive and facilitated diffusion (Mansfield et al., 2010; Kolaczkowska and Kolaczkowski, 2016). Additionally, the discovery of a trisomy in the *ERG11*-containing chromosome in a *C. krusei* strain suggests aneuploidy may not be uncommon in this species, though the effects as it relates to azole resistance are not yet known (Lamping et al., 2009).

Resistance mechanisms against other azoles are also not clearly defined. For example, analysis of itraconazole-resistant *C. krusei* isolates revealed that reduced intracellular content of the drug and not altered affinity for the drug target likely drives itraconazole resistance (Marichal et al., 1995; Venkateswarlu et al., 1996). However, more recently it has been suggested that overexpression of genes encoding both Erg11p and the efflux pump Abc2p may also play a role with itraconazole resistance (Tavakoli et al., 2010; He et al., 2015). Despite its fungicidal activity in *C. krusei* (Rubio et al., 2005), resistance to voriconazole has also emerged, and current research supports a theory where overexpression of the genes encoding the efflux pump Abc2 and Erg11 impart more transient resistance properties, while increased expression of Abc1p and point mutations in *ERG11* predominate as time progresses to yield a stably resistant pathogen in the prolonged presence of voriconazole (Ricardo et al., 2014). Erg11p amino acid substitutions have been observed in azole-resistant *C. krusei* and, in the case of Y166S, have been predicted to interfere with Erg11p function (Ricardo et al., 2014; Silva et al., 2016). While the newer antifungal agents posaconazole and isavuconazole have shown good activity against *C. krusei* (Lee et al., 2000; Rybak et al., 2015), reports of resistance against these agents are relatively sparse (Espinel-Ingroff et al., 2014; Pfaller et al., 2015). However, in a recent analysis examining NAC strains in the U.S. by region, *Candida krusei* resistance to posaconazole was highest in the eastern United States, with posaconazole resistance occurring in 13–16.7% of isolates (Pfaller et al., 2014b). Nevertheless, the mechanisms of resistance in *C. krusei* against these agents remain to be investigated.

### Candida glabrata

*C. glabrata* is unique among the *Candida* species discussed here as it is a haploid yeast more closely related to *S. cerevisiae*. Development of azole resistance in clinical isolates of *C. glabrata* has been almost exclusively linked to the presence of activating mutations in the zinc cluster transcription factor Pdr1 (Vermitsky and Edlind, 2004) that lead to differential expression of downstream targets. Nearly all clinical isolates have been found to have *PDR1* mutations, with such mutations found in the inhibitory domain, activating domain, middle homology region, and xenobiotic binding region. The rapid acquisition of *PDR1* mutations could be due to the high incidence of mutations in the mismatch repair gene *MSH2*, which results in a hypermutable phenotype (Healey et al., 2016). The activating mutations exhibit distinct expression patterns of the downstream effector genes, with the exception of increased expression of *CDR1* and *PUP1*, and no correlation has been found between location of the mutation and altered gene expression (Tsai et al., 2006, 2010; Ferrari et al., 2009; Caudle et al., 2011; Paul et al., 2011). Among the genes whose pleiotropic drug response element (PDRE) is directly bound by Pdr1 (Paul et al., 2014), only three, the ABC transporters *CDR1* (Sanglard et al., 1999), *PDH1* (*CDR2*) (Miyazaki et al., 1998; Sanglard et al., 2001), and *SNQ2* (Sanguinetti et al., 2005; Torelli et al., 2008), have been linked directly to azole resistance. Recent work has shown increased expression of four MFS transporters in clotrimazole resistant isolates compared to clotrimazole susceptible clinical isolates. Disruption of one of these, *TPO3*, moderately increased susceptibility to clotrimazole and fluconazole (Costa et al., 2016). These findings suggest MFS transporters may have a minor role in azole resistance in *C. glabrata*.

Surprisingly, *ERG11* does not appear to play an important role in clinical azole resistance in *C. glabrata* (Sanglard et al., 1999; Vermitsky and Edlind, 2004; Sanguinetti et al., 2005). Increased expression of *ERG11* has been observed in only two clinical isolates of *C. glabrata* (vanden Bossche et al., 1992; Redding et al., 2003). The upregulation in one isolate was later found to be due to duplication of the entire chromosome containing *ERG11* and the phenotype was lost with subsequent passaging in azole-free media (Marichal et al., 1997). A single resistant clinical isolate of *C. glabrata* has been shown to have a nonfunctional 14-α-sterol demethylase due to a missense mutation in *ERG11*, which led to the complete absence of ergosterol in the cell membrane (Hull et al., 2012). No additional clinical isolates have been identified to have resistance mechanisms related to the azole target.

*C. glabrata* has the ability to grow with altered cell membrane sterols, which allows for evasion of azole treatment. *C. glabrata* is able to take up exogenous sterols (Nakayama et al., 2000), both when the ergosterol biosynthesis pathway is blocked and under normal conditions in wild type strains (Tsai et al., 2004; Bard et al., 2005). Aus1p has been identified as the sterol transporter responsible for tolerance to azoles in the presence of exogenous sterols (Nakayama et al., 2007). *C. albicans* has recently been shown to take up sterols under aerobic conditions; however, *C. glabrata* is more liberal in its ability to take up sterols and does so in both aerobic and anaerobic conditions and, in the presence of serum and fluconazole, enhances uptake under aerobic conditions (Zavrel et al., 2013).

Azole resistance in *C. glabrata* has also been attributed to the formation of petite mutants, which are cells that have lost mitochondrial function resulting in respiratory deficiency (Defontaine et al., 1999; Brun et al., 2003). Petite mutants can be generated in the laboratory by treatment with azoles or ethidium bromide. This mutant phenotype has been recovered clinically (Bouchara et al., 2000; Ferrari et al., 2011), but is not common among clinical isolates. Azole resistance in petite mutants has been attributed to upregulation of the ABC transporters *CDR1, CDR2*, and *SNQ2* (Sanglard et al., 2001; Ferrari et al., 2011), which is dependent on Pdr1 (Tsai et al., 2006). Petite mutants exhibit altered sterol profiles with a disproportionate amount of ergosterol and very little of ergosterol intermediates; however, no changes in the sequence of *ERG11* or its expression have been detected (Brun et al., 2004).

## Conclusions

*Candida* species are responsible for a majority of superficial and disseminated fungal infections in humans. While azole antifungals have long provided effective treatment for such infections, recent epidemiological studies indicate that intrinsic azole resistance in some *Candida* species as well as development of high-level azole resistance is a problem of critical importance in the clinical setting. While extensive studies to elucidate molecular mechanisms of high-level azole resistance in *C. albicans* has uncovered the role of ergosterol biosynthesis gene mutation and ERG gene and drug efflux pump upregulation as key mediators of azole resistance, there are clearly other factors at play that contribute significantly to such resistance. Similarly, while NAC are closely related to *C. albicans*, that does not necessarily translate to analogous molecular mechanisms of azole resistance.

Of the NAC species highlighted in this review, *C. parapsilosis, C. tropicalis, C. krusei*, and *C. glabrata* all express ABC transporter and/or MFS genes orthologous to *CaCDR1* and *CaMDR1*. However, as discussed, the altered expression of these genes in azole-resistant NAC appear to contribute differently to resistance in different species. Moreover, the transcriptional regulators and genetic mutations governing azole efflux and sterol biosynthesis in *C. tropicalis, C. parapsilosis*, and *C. krusei* have not been fully examined. Finally, there exist clear differences in the mutations in *ERG11* that are found to influence azole resistance in clinical isolates among these species. As azole resistance continues to emerge in these species, a more complete understanding of the important differences among resistance mechanisms employed by these species will be needed in order to circumvent this important clinical problem.

## Author contribution

SW wrote the section regarding _*Candida glabrata*_, contributed to the Introduction, and edited the overall text. EB wrote the section regarding _*Candida parapsilosis*_ and contributed to the Introduction. JR wrote the section regarding _*Candida tropicalis*_, contributed to the Introduction, and compiled the data in Table 1. AN wrote the section regarding _*Candida krusei*_, contributed to the Introduction, and designed Figure 1. KB wrote the section regarding _*Candida albicans*_, contributed to the Introduction, and edited the overall text. PDR oversaw the review, wrote the abstract and conclusion, contributed to the Introduction, and edited the overall text.

## Funding

Research from the laboratory of PDR is funded through a grant from the National Institutes of Health (R01 AI058145).

### Conflict of interest statement

The authors declare that the research was conducted in the absence of any commercial or financial relationships that could be construed as a potential conflict of interest.
